# Plausibility of patient-centred care in high-intensity methadone treatment: reflections of providers and patients

**DOI:** 10.1186/s13722-021-00251-9

**Published:** 2021-06-29

**Authors:** Kerry Marshall, Geoffrey Maina, Jordan Sherstobitoff

**Affiliations:** 1 University of Saskatchewan, Saskatoon, Canada; 2 Selkirk College, Castlegar, Canada

**Keywords:** Opioid use disorder, Health care providers, Canada, Methadone, Buprenorphine/naloxone, Treatment outcomes, Social justice, Patient-centred care

## Abstract

**Background:**

Patients with opioid use disorder (OUD) often have complex health care needs. Methadone is one of the medications for opioid use disorder (MOUD) used in the management of OUDs. Highly restrictive methadone treatment—which requires patient compliance with many rules of care—often results in low retention, especially if there is inadequate support from healthcare providers (HCPs). Nevertheless, HCPs should strive to offer patient-centred care (PCC) as it is deemed the gold standard to care. Such an approach can encourage patients to be actively involved in their care, ultimately increasing retention and yielding positive treatment outcomes.

**Methods:**

In this secondary analysis, we aimed to explore how HCPs were applying the principles of PCC when caring for patients with OUD in a highly restrictive, biomedical and paternalistic setting. We applied Mead and Bower’s PCC framework in the secondary analysis of 40 in-depth, semi-structured interviews with both HCPs and patients.

**Results:**

We present how PCC's concepts of; (a) *biopsychosocial perspective*; (b) *patient as a person*; (c) *sharing power and responsibility*; (d) therapeutic *alliance* and (e) *doctor as a person*—are applied in a methadone treatment program. We identified both opportunities and barriers to providing PCC in these settings.

**Conclusion:**

In a highly restrictive methadone treatment program, full implementation of PCC is not possible. However, implementation of some aspects of PCC are possible to improve patient empowerment and engagement with care, possibly leading to increase in retention and better treatment outcomes.

## Background

Methadone and buprenorphine/naloxone (suboxone) are two medications commonly used to treat opioid use disorders (OUD). In Canada, up until recently, methadone has been the treatment of choice for managing OUD through methadone treatment programs [1]. However, compared to buprenorphine/naloxone, methadone has a high-risk profile for overdose. To mitigate the risk for overdose with methadone, health care providers (HCPs) use direct daily observation of methadone ingestion and routine toxicology screens to monitor compliance with the treatment [2]. This involvement in patients’ care is contested as a form of governmentality, where methadone treatment is deemed a technology that is meant to produce productive and obedient subjects [3, 4]. Restrictive methadone treatment programs informed by this governmentality often have imbalanced power dynamics where HCPs play the role of disciplinarians and enforcers of the desired code of conduct [3, 5, 6]. Patients' powerlessness in contributing to treatment related decisions is exacerbated by diverse social and health inequalities and complex medical conditions, which may impede a patient’s ability to follow through with the treatment contract [5, 7–9].

Besides the restrictive nature of methadone treatment, patients receiving care often experience stigma from society and HCPs, based both on their substance use and for their enrollment in methadone treatment [9–11]. Stigma from HCPs impedes access to care and positive clinical outcomes [10, 11]. Furthermore, retention of patients on methadone treatment varies and is dependent on multiple factors. These factors include equitable access to social services, employment, carry privileges, positive interactions between HCPs and patients, family and other social support, and positive attitudes towards methadone treatment [12].

Social justice involves equitable redistribution of power and resources amongst all members of society [13]. Resources consist of anything that may have a positive effect on health, including social support and access to services that are inclusive, equitable and just [14]. Patient centered care (PCC) reorients care to focus on essential outcomes to the patient and creates space for the patient’s to be engaged as equals, all while acknowledging them as individuals with unique values, experiences, and goals [15–17]. Because patients on methadone treatment are often socially marginalized, they lack social support and advocates [6]; therefore, HCPs have moral and ethical obligations to advocate and support patients in their treatment journey regardless of their conditions [18, 19], PCC is a beneficial framework when working with this community. PCC framework contradicts the paternalistic biomedical model of care because it considers patients' preferences or wishes [20]. Paternalism in patient care creates challenges to advocate for patients best interests and may perpetuate biases caused by sex, race, culture, and socioeconomic status, affecting decision making; therefore, depriving the patient of the opportunity to make decisions that reflect the reality of their lived experience, condition and preferences [21].

A PCC framework is congruent with principles of social justice in health, such as access, equity, participation and human rights, which are espoused in PCC’s mantra of “no decision about me without me” which guides clinical practice and patient-provider engagement [22]. Social justice and PCC parallel each other and work together. First, when practicing within a framework of social justice, HCPs are ethically obligated to act against forms of inequity and oppression [13]—including within their own practice. Therefore, HCPs may be compelled to modify their practice to ensure that patients are treated with respect, dignity and provided with opportunities to contribute to decisions regarding their care [13], paralleling the goals of PCC. Secondly, applying social justice principles in methadone treatment care may commit HCPs to collaborate with patients in their care, irrespective of their social capital and health disparities [23]. Thirdly, implementing social justice principles when working with priority populations is imperative as not understanding the importance of social justice can decrease patient satisfaction, treatment adherence, and health outcomes [24]. Lastly, applying social justice in health care may also mean confronting stereotypical portrayal of patients on methadone treatment as lacking agency and unable to actively engage in their care [6, 25].

## Methods

This study is based in a Canadian mid-sized prairie province. The setting from which the project was based operates as a specialized clinic, serving approximately 350–400 patients from neighbouring communities. The specialized nature of this clinic means that they only receive care for OUD and must seek primary care services and adjunct support elsewhere. Patients access daily MOUD from methadone dispensing pharmacies that are distributed across the city. Although buprenorphine/naloxone is the recommended drug of choice for treatment of OUD, most patients are on methadone. Patients can be switched to buprenorphine/naloxone from methadone and vice versa based on clinical outcomes, ability to tolerate the medication, and socioeconomic considerations.

The clinic comprises of a physician, methadone case coordinators, laboratory technician and pharmacy technician and pharmacists working in methadone dispensing pharmacies. Patients access the clinic by self-referral or through referral by a HCP. During an initial visit to the clinic, a comprehensive assessment is conducted to determine their enrollment suitability. As this program is biocentric in nature, HCPs are given immense power to determine if the patient would be initiated in or terminated from treatment. Once patients are formally enrolled in the treatment, they sign a treatment agreement which stipulates conditions they must follow to maintain their enrollment in the treatment program. Again, as the clinic is based on a biomedical, highly restricted care model, abstinence from substance use when enrolled in treatment is expected and enforced. Incompliance to treatment contract threatens their retention to care.

The majority of patients from neighbouring communities use medical taxis to access the clinic. In the community, patients on MOUD experience stigma rooted in the misunderstanding that methadone is addictive and harmful to their health. Since there is no direct collaboration between the community health center and the methadone treatment clinic, these two settings do not leverage their resources to support the patients. Housing shortages and persistent substance use in patients’ home communities often mean that patients on MOUD do not have safe recovery environments. With the disintegrated care system and lack of coordination between the community health center and the methadone treatment clinic, the possibility of individualized, holistic, and equitable care may not be available for patients that require comprehensive wraparound care.

In this secondary analysis, we aimed explore how HCPs were applying the framework of PCC for patients on methadone treatment based on the biomedical model of care. Although multiple frameworks for PCC exist, we were drawn to Mead and Bower’s [17] framework to help us make sense of PCC in a highly restrictive model of OUD care. Mead and Bower’s [17] framework therefore informed the secondary analysis of two datasets involving experiences of HCPs and patients in provision and receiving of MOUDs, respectively. The following research question informed the analysis: are PCC principles applicable in methadone treatment centers? Using both the perspectives of the HCP and the patient, we deeply understood the relevancy and applicability of PCC in this setting (Table 1).

**Table 1:** Sociodemographic characteristics of patients

Sociodemographic characteristics				
Gender	Female		Male	
Characteristic	Frequency	Percentage (%)	Frequency	Percentage (%)
Age				
Under 30	2	15.4	–	–
30–39	3	23.1	8	88.9
40–49	8	61.5	1	11.1
Marital status				
Partner	8	61.5	6	66.7
No partner	2	15.4	–	–
Not mentioned	3	23.1	3	33.3
Level of education				
< grade 10	1	7.7	1	11.1
Grade 10	6	46.2	4	44.4
> grade 10 ≤ grade 12	3	23.1	3	33.3
Post high school education	2	15.4	1	11.1
Ethnicity				
First Nations	13	100	8	88.9
Other	–	–	1	11.1
Employment				
Employed	–	–	1	11.1
Unemployed	12	92.3	7	77.8
Not mentioned	1	7.7	1	11.1

In the primary research, we used an exploratory qualitative study design to examine the experiences of HCPs and patients in the provision and receiving of care. Convenience sampling was used to recruit patients to the study. GM visited methadone pharmacies and circulated recruitment flyers. Patients interested in participating in the study contacted GM by phone to arrange for an interview. In the community, the clinic methadone case coordinator assisted GM with recruitment logistics. A day was set to come to the community to interview patients once there was a sizeable number of participants who signed up for the study. Convenience sampling was applied in the recruitment of the HCPs at the clinic. GM sought appointments with HCPs to explain the purpose of the study. Those that consented were formally enrolled and interviewed within the precincts of the clinic. To be enrolled in the study, patients were to be at least 18 years old, able to consent, and enrolled in the methadone treatment program for at least six months. HCPs with at least six months of experience providing care to patients on MOUD were invited to participate in the study.

Forty individual interviews, lasting on average twenty-five minutes, were digitally recorded and transcribed verbatim with eighteen HCPs and twenty-two patients. A semi-structured interview guide was the main mode of facilitating the interviews with the patients which focussed on (a) substance use history; (b) experiences with methadone treatment; (c) access to treatment; (d) adherence to treatment; (e) support available; (f) mental health and (g) challenges faced in treatment. The focus of the interviews with HCPs was (a) role at the clinic; (b) experiences providing care to patients with OUD and (c) barriers to providing optimum care. Two research assistants separately recoded and analyzed the two data sets as two projects. A third person reviewed the codes that were developed by the research assistants and harmonised them to form a coding framework that was informed by the PCC framework. Thematic analysis was applied to the data analysis using the following steps as identified by Kiger and Varpio [26], which entail (a) familiarizing with the data by reading the interviews at least two times; (b) generating initial coding of important ideas, constructs that relate to the Mead and Bower’s [17] overarching components of PCC; (c) searching for themes—which involved consolidating nodes that were related to the five components of PCC and; (d) reviewing the themes to make sure that there were sufficient and strong excepts to communicate the extent to which the five components of PCC were applicable in the care of patients on MOUD. Before the commencement of the study, Ethics Certificate (BEH 44-17) was obtained from the University of Saskatchewan Ethics Review Board (Table 2).

**Table 2:** Summary of health provider characteristics

Healthcare providers	no	Experiences in the clinic
Methadone Case coordinators	4	1–14 years
Physicians	4	1–4 years
Pharmacists and pharmacist technicians	6	6 months to 19 years
Laboratory technologists	1	11 years
Managers	2	1–3 years

## Results

In this section, we present the study's findings on the experiences of HCPs and patients in methadone treatment, focusing on the extent to which the methadone treatment program engages in PCC. We use the Mead and Bower [17] framework that identifies five facets of PCC; (a) a focus on biopsychosocial perspective; (b) considering “patient as a person”; (c) sharing power and responsibility; (d) developing a therapeutic alliance and; (e) applying the principle of the meaning of “doctor as a person”. We completed secondary analysis deductively, based on the chosen PCC framework. See table three for quotes and exploration of themes summarized in findings section.

### A focus on biopsychosocial perspective

The biopsychosocial perspective emphasizes understanding the person as a whole being, which allows the needs of the patients to be met holistically. HCPs conducted full assessments of patients at the initial enrollment into methadone treatment to determine the program's care needs and patient suitability. HCPs caring for patients on MOUD are acutely aware of the complex physical, mental, and psychological issues patients present due to chronic polysubstance use. These complexities include diabetes, HIV, hypertension, malnutrition, and concurrent disorders such as depression, anxiety, post-traumatic stress disorders.

*[When patients come to enroll for treatment] we interview the patients first and do a full bio, social, psycho[logical], and spiritual assessment. The report is then given to the doctor, who decides if the patient is a good candidate to be enrolled in the program. Once enrolled, we start moving them through the stages of recovery* (Methadone Case Coordinator 1)*.*

With this assessment, HCPs can anticipate patients' health care needs and factors that may affect treatment and recovery, like histories of sexual, physical, and verbal abuse; concurrent health conditions; and lack of social stability. Since most patients sought treatment after a chronic substance use period, their treatment and recovery were understandably complex. Describing the complexity of their condition, one HCP noted:

*They're [The patients in our program] very complex… and we know that [late stage] drug use, there isn't an area that hasn't been touched in their lives. Their health has been [affected], their children have been taken away, they're in the correctional system, they don't have a home... so yeah. They're very complex because there is mental health issues, [and] there are health issues…* (Methadone Case Coordinator 1)*.*

Despite the complex needs of the patients, the clinic setup did not provide comprehensive care to them. Therefore, patients sought primary and adjunct care services for their concurrent conditions on their own, a task that was made difficult by lack of care navigators. Moreover, since methadone treatment patients were deemed “'too complex” to care for, they struggled to find a family doctor.

*What we've been noticing is that primary health care physicians don't want to take these patients as patients because they're so complicated. Prescribing methadone physicians don't want to be their primary healthcare provider either. But I think if really, if we want to look at holistic care, we need to provide care to these patients for their other conditions as they have chronic diseases and other social needs that need to be addressed (Manager,* 1).

Most patients understood the gravity of their medical condition and their challenges in recovery due to chronic substance use. Despite their commitment to recovery, patient’s lacked safe housing options where they would not be tempted to use substances. Nevertheless, they were well aware of what they needed to do to get their lives back (Table 3).

*"I think I need to get my place back and get back into school and stay away from these people… stop going around their places"* (Female patient, 39 years).

**Table 3:** Summary of quotes and themes

Component of PCC	Quote	How it facilitates PCC	How it does not support PCC (Barriers)
Biopsychosocial perspective	*When patients come to enroll for treatment, [we] interview them first and do a full bio, social, psycho[logical], spiritual assessment. The report is then given to the doctor, who decides if the patient is a good candidate to be enrolled in the program. Once enrolled, we start moving them through the stages of recovery* (Methadone Case Coordinator)	Comprehensive assessment of patients during intake allows for a deeper understanding of the patients physical, mental and social needs	Since the clinic provides specialist care, patients have to seek care for the identified needs elsewhere
Patient as person	*You give them their drink, and you might have a little bit of a conversation. They [the patients] might be, you know, like one gal said, 'Oh I walked over here today and, you know, I'm trying to get back into- I used to run and,' so you learn a little bit about their lives and, you know, as you interact with them, one guy that is a drywaller so I was asking him is it busy right now?' He said, 'Yeah I got lots of drywall jobs,' so you learn a bit personal about them* (Pharmacist)	HCP interest in understanding a patients’ lived experience motivates HCPs to be advocates and allied in care	There are limits to what HCPs can do for these patients who are dictated by treatment guidelines that governed the clinic operationalization
Sharing power and responsibility	*I was taking Suboxone at the time, and then, I started getting depressed, really depressed inside. I would take Suboxone, and I'd pretty much sleep for three days or four days. So, I went back, and I talked to her [the methadone case coordinator] about it, I said, "This is not working." She was wondering because I quit taking Suboxone. I told her, "Well, do you think we can try methadone?" And she's like, "Okay." That was my first round with methadone* (Female patient, 38 years old)	When patients are able to contribute to their treatment plan, they may be more successful in treatment adherence and recovery	A significant power imbalance between HCPs and patients exists, which had a significant bearing on patients' ability to discuss their treatment goals
Therapeutic alliance	*If we need any help in basically anything like we need somebody to talk to or job, resume hunting or something… 'Cause I needed a little bit of money last week for my daughter's graduation gift so they hired me to do some cooking. I went in there and cooked them a quick couple meals and boom, gave me some money. So if I need some stuff like that, they help out…they help out a lot. These two ladies are very well, they do a good job* (Male patient, 38 years old)	Patients benefited from positive and mutually respectful encounters with HCPs as it makes patients feel valued	A lot of time is needed to develop therapeutic alliance between the patient and the HCPs. Noncompliance with the treatment plan is a significant threat to the therapeutic alliance
Doctor as a person	*I've never taken things personally [with patients on* methadone treatment*]. It's gotta be professional. And with my prior careers too and stuff, I've always been able to deal with situations well. It's never affected me mentally or emotionally. And that's the type of person I am. How I've seen it affect people—death has affected people. I've seen that. I've seen death numerous times, and never affected me. So, in a case where these people are, it's professional, right? One patient got emotional yesterday, and I feel for her, but it just doesn't affect me* (Methadone Case Coordinator)	Because of complexities involved in the caring of patients with OUD, objectivity in care can be protective to both the patient and the provider	Some patients may feel the boundaries set by HCPs and the treatment contract acted as barriers to achieving full recovery, ultimately hindering the ability to form a therapeutic alliance

Although HCPs understand that patients seeking MOUD needed holistic care, the lack of resources and holistic care threatened patients' ability to recover from OUD.

### The “Patient-as-Person”

The concept “patient-as-person” encompasses the individual's understanding, experiences, and derived meaning of their illness [17]. Patients in this study are acutely aware of the impact that substance use has on their lives, the need for treatment, and the unique challenges they are likely to face during the treatment and recovery. This insight often motivated patients to change their behaviours and aided in an understanding of the complexity of their recovery journey.

*When I first started off, it was just alcohol. For that, I started off with the drinking when I was a kid, and I was drinking until... I drank heavily for years, and years, and years until about 2013, 2014. That's actually the last time I drank, so I drank heavily with that. But all along the way, I was doing stuff like cocaine, crack, I smoked weed a lot during the time, I did a lot of that. I did pretty much anything a guy could get high on and I did a lot of that, minus the hairspray and stuff like that, there were certain things I wouldn't do. But anyway, I had [done]a lot do drugs in my lif*e (Male patient, 38 years old).

Patients’ lived experiences grounded them in their recovery journey and aided their personal goals. Through these experiences, patients clarified essential issues in their lives -especially the need to improve or restore broken relationships with their families. Having witnessed many fatal impacts of substance use, such as losing loved ones, some used such tragedies as motivation to seek treatment and change their life course. Nevertheless, the motivation to enter treatment does not take away the enduring personal pain caused by the impact of substance use, both at the individual and community levels.

*…Because I'm scared. I'm tired of feeling pain all the time. I'm scared of the withdrawal for one, and I'm scared that the withdrawal is gonna be so bad for me. I'm scared I'm gonna... [Crying] Sorry… I'm scared that I'm gonna end up committing suicide. I look out my apartment window and I see the rivers, the trees, the bridge. I don't want [my husband] to know, but I've thought of hanging myself so everybody can see me… I'm scared of withdrawal, and pain, and you just want it all to go away* (Female patient, 42 years old)*.*

When HCPs address a patients’ pain, they attain a more in-depth insight into the root cause of substance use and the complexities of SUD. Undoubtedly this creates a window to view the patients for who they are as individuals.

*You give them their drink [of methadone], and you might have a little bit of a conversation… so you learn a little bit about their lives and, you know, as you interact with them. One guy that is a drywaller, so I was asking him “is it busy right now?” He said, “Yeah I got lots of drywall jobs,” so you learn a bit personal about them* (Pharmacist 1)*.*

A more profound interest in knowing a patient’s lived experience motivated HCPs to be their advocates and allies in care. However, there are limits to what HCPs can do for patients as care is dictated by strict treatment guidelines.

### Sharing power and responsibility

Sharing power and responsibility is characterized by a balance of power with mutual participation in treatment and encouraging the patient to assume an active role in their care [17]. With this concept, the patient’s needs and preferences are heard and respected, and they are actively involved in decision-making. As mentioned, patients enrolled in methadone treatment must sign a non-negotiable treatment contract that outlines their expected behaviours. These behaviours include abstaining from substance use during treatment, providing a urine sample on request for toxicology, and reporting daily to a pharmacy to take their methadone doses under observation. If patients fail to adhere to the treatment, they risk termination from the program or HCPs tapering their MOUD dose. It is apparent that the power to enroll or discharge a patient from the methadone treatment program solely rests on the HCPs power and judgement. Patients took on the responsibility of their recovery by voicing concerns to the HCP team. When patients can contribute to their care plan, they may be more successful in treatment adherence and recovery.

*I was taking Suboxone at the time, and then, I started getting depressed, really depressed inside. I would take Suboxone, and I'd pretty much sleep for three days or four days. So, I went back, and I talked to [the methadone case coordinator] about it, I said, "This is not working." She was wondering because I quit taking Suboxone. I told her, "Well, do you think we can try methadone?" And she's like, "Okay." That was my first round with methadone* (Female patient, 38 years old)*.*

Although this patient was comfortable advocating for themselves, not every patient felt they had the confidence to provide treatment suggestions to their HCP that could enhance clinical experiences. Our findings show that the most common treatment request from the patient was a dose increase or getting carry privileges or “carries.” Getting carries would save them from daily trips to the pharmacy for medication pickups. Daily trips create many treatment barriers for individuals who lack transportation or live rurally. Patients also verbalized the need to increase their dosage as the current dose was inadequate to address their withdrawal symptoms and cravings. Persistent withdrawal and cravings—even when on methadone—increased the risk of substance use, inadvertently contravening the treatment contract and delaying their care progress.

*[The doctor] says I have to wait [for dose adjustment] because I have to keep taking my methadone, but I keep on missing [taking the medication] because I don't have the money, and every time I miss… if I miss so much, then I don't go up [in dose]. I have to wait 'til I have like seven drinks and then move up, but I keep on missing because I don't have the money to get it so it's taking a while* (Male patient, 39 years old).

Although HCPs acknowledged that treatment success depended on the patients’ behaviour, it was evident there was a power imbalance between HCPs and patients, which had a significant bearing on patients' ability to advocate for their treatment goals. HCPs felt strongly responsible for their patients' health because they understood the gravity of substance use when patients were on MOUD. Therefore, if HCPs believed that patients may be using substances, they did not hesitate to approach the patient or order toxicology screening. This HCP belief may be nuanced where, although discontinuing the contract risked causing patients to relapse, it saved them from potential overdose and death.

### Developing a therapeutic alliance

Therapeutic alliance focuses on personal relationships between HCPs and patients [17]. According to Mead and Bower [17], patient-centeredness emphasizes priority of personal relationships between HCPs and patients, and embracing empathy, congruence, and caring to achieve an effective therapeutic change in patients. For the most part, HCPs made every effort to communicate support to the patients by accepting them for who they were. However, developing a patient-provider relationship can take time.

*I just do a lot of encouragement and just like we believe in you, we love you [laughs] you know. I've known them for ten years. Like one patient I said “man we could be sisters. I've seen you in your good times, I've seen you in your bad times, I've seen you grouchy, [laughs] dope sick”, I've seen just because I've worked in so many like see them on the street, see them in the clinic, see them you know. So really, at the end of the day, I think the biggest thing is a relationship* (Methadone Case Coordinator 2)*.*

In building an effective therapeutic relationship, every recovery step—however small—is celebrated. This relationship is fostered by the patients' desire and determination to show active engagement in their care and in everyday activities that demonstrate their agency as “productive” members of society.

*If we need any help in basically anything like we need somebody to talk to or job, resume hunting or something... 'Cause I needed a little bit of money last week for my daughter's graduation gift, so they hired me to do some cooking. I went in there and cooked them a quick couple meals and boom, gave me some money. So, if I need some stuff like that, they help out…they help out a lot. These two ladies are very well, they do a good job* (Male patient, 38 years old)*.*

Building a therapeutic alliance with the patients is vital as it made patients feel valued, inspired and promoted a positive attitude towards their treatment. Trust building was enhanced when patients were honest, consistent and adhered to treatment contracts. HCPs were then able to advocate for patients as required.

*I had a guy that he, he just started a new job that was a Monday to Friday kind of like 8:30 to 6:00 job like he was doing trucking. So, he couldn't, he legitimately couldn't make it into the store, and it was a new job for him, he was trying to do really good, trying to better himself, and so I talked to the doctor and recommended that we switch to like a Saturday pickup so that he could then make it to work every day and you know he was a patient that was doing pretty well before so it was something that I recommended to the doctor and the doctor agreed because we need to, we need to work with patients* (Pharmacist 2)*.*

HCPs required patience to build therapeutic relationships with the patients. New patients often struggled to appreciate the necessity of the rules of the treatment contract and, as such, needed to be socialized on boundaries and treatment requirements.

*No, I'd say the regular patients are not that- they're not challenging at all. When we get some new patients, sometimes there can be challenges. It's almost like kind of getting them to getting to know where their boundaries are, right? And if they had a much bigger boundary before, they usually get reeled in pretty quick. So that's a challenge. Just for them to know what's acceptable and not acceptable at our place, right? How much running we're going to do for them, and how much we're not* (Pharmacist 1)*.*

Even with boundaries that appeared to be challenging to navigate, patients benefitted from positive and mutually respectful encounters with HCPs, which accelerated the development of the therapeutic alliance. Supportive HCPs made a tangible and positive impact on the patients.

*Well, the system, the Suboxone program, yeah, it is a big help, actually. I was going through some stuff here the past month and the coordinator, their doctor, really helped me through it, and he was really helpful* (Female patient, 32 years old)*.*

### Applying the “Doctor as a Person” concept

The “doctor-as-person” concept addresses the HCPs subjectivity inherent in the patient-patient relationship [17]. This dimension calls upon the HCP to reflect and have self-awareness of emotional responses that may influence the patient-HCP relationship. In this study, it was evident that the patients' experiences with substance use, ability to adhere to treatment contracts, and commitment to recovery significantly influenced the HCP's perception of the patient.

Some HCPs detach themselves from being influenced by the social realities that patients face, and solely focus on the treatment guideline as the basis for a therapeutic alliance. HCPs deemed rigidly following the treatment guidelines as necessary because it ensures patients' safety from overdosing, especially if they continue using substances. Using the treatment guidelines allows the HCP to be objective in the therapeutic encounter, even though encounters may come across as cold and non-supportive.

*I've never taken things personally [with patients on methadone treatment]. It's gotta be professional. And with my prior careers too and stuff, I've always been able to deal with situations well. It's never affected me mentally or emotionally. And that's the type of person I am. How I've seen it affect people—death has affected people. I've seen that. I've seen death numerous times, and never affected me. So, in a case where these people are, it's professional, right? One patient got emotional yesterday, and I feel for her, but it just doesn't affect me* (Methadone Case Coordinator 3)*.*

HCPs reported the need to set boundaries when caring for patients on MOUD. At times, these boundaries come across as blunt and emotionless, but provide HCPs with a means to stay professional when working with patients. Boundaries protect HCPs by helping them abide to the treatment guidelines, even if it means they are seen as rigid in their patient interactions Patient’s engagement with care can nonetheless influence the way the providers perceive them.

*There's some patientele that I enjoy working with. And there's some patientele I don't usually, because they [the patients] don't do anything for themselves, right? So, for the ones that are proactive and wanting to legitimately better themselves, I enjoy working with them* (Methadone Case Coordinator 3)*.*

Some patients may feel the boundaries and treatment contract set by HCPs acted as barriers to achieving full recovery, ultimately hindering the ability to form a therapeutic alliance. But patients were also aware that some interactions with HCPs might be informed by prior experiences with patients, both positive and negative.

*[when talking about carries] the doctors are really just… I can't explain it. Maybe they had some really bad experiences that makes them… I don't know, I don't wanna judge them because I don't know. But I think there's a really big lack of education, big time….* (Female patient, 43 years old).

Over time, patients got used to the treatment structure set in place to guide their recovery. With an understanding of the rationale for the treatment approach, patients were more cooperative and did not have hard feelings towards HCPs, especially when their requests were not considered.

*No, no. Actually, the staff out here has been pretty good. They [the staff] actually come to me with questions wanting to talk so they've been pretty good* (Male patient, 31 years).

Despite the boundary setting that can appear rigid, HCPs celebrated with the patients when they made progress in their treatment journey. This rejoicing recognized the hard work that both the patient and the provider had invested in the patients' recovery journey.

*Success can be a number of things. Sometimes it can be as little as harm reduction, whereby instead of using substances fourteen times in two weeks, maybe they've used it four times. It can be making progress in their recovery whereby at the time of enrollment in the program, all they had was a backpack. And then, a month or two later, they found stable housing, and then they're working on going to get their children back. Like this is the most rewarding career in terms of seeing the changes that happen so quickly in patients' lives* (Methadone Case Coordinator 4)*.*

Setting boundaries between the patient and the HCP allows practitioners to be objective in their therapeutic encounters. Although developing a trusting therapeutic relationship took time, helping patients understand the rationale behind their treatment structure was positive in their recovery journey.

## Discussion

We explored the extent to which the framework of PCC is applicable and relevant in highly restrictive methadone treatment programs. As a specialized clinic, the methadone treatment program is based on the biomedical model, which creates limits for HCPs to accommodate the patients' unique desires and needs. Thus, there are limitations to the extent PCC can be incorporated within the current, highly restrictive methadone treatment programs.

Comprehensive assessment of the patient’s condition and treatment preferences at enrollment to the methadone treatment program allows for planning and mapping wraparound services that are important to the patient [27]. The information gathered during the initial assessment could provide an opportunity for HCPs to anticipate the patients’ recovery trajectory and collaboratively plan ways to care and support the patient in their recovery. However, HCPs often determine patients' suitability for the program based on clinical markers stipulated in the practice guidelines. When HCPs do not have a clear understanding of patients' needs and goals or their lived experiences, it may perpetuate negative attitudes towards those who use substances. This may have a negative impact on patient engagement with care and treatment outcomes [28].

Clinical settings with comprehensive services tend to have an increased retention to care and positive treatment outcomes compared to those that don’t [29, 30]. Patients were more motivated to engage and remain in treatment if the treatment addressed their mental, physical and social health, and the interventions assisted them to be more functioning in society [31]. The lack of comprehensive services for those on MOUD presents a missed opportunity to incorporate PCC within treatment, as there were limited ways from initiation for patients to be actively engaged in their care [30].

As a biomedically oriented clinic, the methadone treatment program does not provide opportunities for shared decision-making power between patients and the HCPs. As patients are motivated to engage and remain in treatment to become fully functioning society members if their needs are met [31]. Sharing decision-making privileges with the patients can have tremendous impacts on their clinical outcome, including better quality of life from social, emotional, and mental domains [32]. Yet, to apply a PCC framework, HCPs are required to holistically consider the unique circumstances that may affect patients' ability to engage with care as per the treatment contract [17]. These circumstances may include the home environment, social support, patient preferences, and relevant socioeconomic variables [17, 33]. As such, current treatment rules and requirements, such as daily pharmacy visits to receive their methadone dose, may prevent patients from reintegrating fully into society, as it may interfere with inflexible work schedules or the ability to travel [34]. Therefore, these restrictions create a barrier to care and cause patients to lose motivation to continue treatment [31, 35].

Patients on MOUD have multiple and complex health needs that require assistance and support to access care. With limited linkage services provided by the methadone treatment clinic, long waiting times and lack of material and social capital resources necessary to navigate care hampered patients' ability to access these services. To foster support for these patients, implementing inexpensive interventions such as peer recovery support services that have been effective in linking patients to required services is needed [31, 36, 37]. Peer-based interventions can include multiple dimensions of support such as mentorship and education, from those who have lived experiences using substances [36]. Peer-based interventions can also provide much-needed support and encouragement to empower patients to take responsibility for their recovery [31, 35–37].

People on MOUD hypothesize that peer-based interventions would play a positive role in their care—from outreach to promote programs to assisting with navigating treatment expectations [31]. In these environments, implementing peer recovery intervention values lived experiential knowledge, can help decrease substance use, and increase treatment adherence [36, 37]. For example, Bernstein [38], found that when participants had a brief motivational telephone call with someone with lived substance use experience, they were more likely to be abstinent from cocaine and heroin after a six-month period. This example highlights how peer-based interventions can be minimal, requested by people on or thinking about MOUD, and increase treatment adherence.

The therapeutic alliance is thought to be an essential PCC aspect [31, 38]. Facilitating strong interpersonal relationships allows patients to feel empowered, have autonomy in their care, and enhance active engagement in treatment [31]. When patients feel respected and appreciated, they are more inclined to remain in treatment [31]. However, if the alliance is not well-formed, patients' experience and recovery can be hampered by unsafe patient-provider encounters that can cause stigma within healthcare systems [34, 39]. Ineffective therapeutic alliance may also result in resentment with patients who can misinterpret treatment contract requirements—such as urine toxicology tests and observation of methadone ingestion—as policing, which was shown within this study. To promote empathy, support, and caring, HCPs need to appreciate patients' social determinants of health and the root causes of inequities faced by patients [13].

Additionally, limited time may be a factor that hinders the ability to build a therapeutic relationship between HCPs and patients. As mentioned by participants, there is limited time resources by the physician to spend with patients attending the methadone treatment program. More quality time spent with patients contributes to higher patient satisfaction and potentially better patient outcomes for those with chronic conditions [40]. Other recommendations to improve time spent with patients to improve satisfaction include having clear and consistent expectations, actively listening to patient concerns, and engaging patients’ in their care [40]. All of these suggestions directly align to with a framework of PCC. Incorporating management of the time spent with patients may require a culture shift within organizations to focus on understanding and valuing the voice and lived experiences of patients.

When HCPs appear overly professional, the patient may inadvertently see them as being superior, ingenuine and untrustworthy which creates challenges when developing a mutually beneficial therapeutic alliance as power is not balanced [41]. With this, professionalism may hinder the ability to form a mutually beneficially therapeutic alliance. Considering there are professional obligations that HCPs have when following treatment guidelines for MOUD, their need to be professional may be heightened considering the potential for internalized stigmatizing beliefs around people who use substances [42]. For example, a common belief is that people who use substances are not to be trusted, which may create tensions between the ability for the HCP to maintain boundaries and be professional, while incorporating patient desires into treatment [42]. Boundary setting for HCPs working with challenging patients can prove to be difficult as tensions exist between the care that HCPs may want to provide, versus the care they are able to provide given the constraints of the health care system and culture within it [43].

The concept of a doctor as a person invites self-reflection and self-awareness of who the HCP is to the patient. This concept also influences the way HCPs relate with patients and their ability to empathize with the patients' condition. Irrespective of the methadone treatment clinic's limitations, patients respond best when HCPs are seen to be caring, supportive, hopeful, empathetic, and genuine in their encounters [27]. Caring HCPs can facilitate trusting relationships, empower patients, and ensure optimal patient care is provided [44]. Furthermore, communicating care to patients can provide an avenue for patients to share their concerns and hopes with the HCPs, and provide input to their care [17]. By practicing both caring and self-awareness, HCPs can attend to any inherent biases, stigmas, or beliefs that may hinder them from communicating positive regard to the patients [17, 39]. Moreover, applying caring concepts can create a mechanism where patients are supported holistically, trusting in their abilities, validating experiences, and being present to their experiences [44].

Fragmented health services, lack of collaboration between the community health center and the methadone treatment, and a biomedical approach to treatment are significant impediments to PCC being fully operationalized [27]. Nevertheless, although the provincial jurisdiction provides the biomedical framework of methadone treatment, possibilities of enhancing PCC can be imagined by reorienting the focus of methadone treatment to patient-centred and recovery-oriented care [27]. Such an approach would privilege patient engagement with care and recovery instead of seeking absolute abstinence from substances while on treatment [27]. Recovery-oriented care emphasizes equitable distribution of services, patient-oriented goals and interventions to increase recovery capital [33]. In this approach, attention is paid to the type of social capital—human relationships, physical capital, economic; human capital, individual attributes; and cultural capital, values, and beliefs available to the patients for recovery [33].

Recovery-oriented care, therefore, focuses on providing support and management at each stage of the recovery process, before initiating treatment, during treatment, and post-treatment [45]. Building recovery capital can include utilizing peer supports, focusing on reducing harm, educating HCPs to understand social justice principles for equitable distribution of resources, understanding historical trauma and the impact of racism on health, and working towards a treatment program rooted in PCC [27, 33, 46].

Shifting the approach to care from restrictive treatment contracts towards building recovery capital can be a starting point at the clinics to encourage recovery orientation [27, 33]. A PCC framework reinforces and overlaps with principles of recovery capital and social justice and can allow for fair distribution of society's benefits and responsibilities and their consequences [47]. Moreover, integrating the framework of PCC and social justice can reorient focus to the root cause of inequities that predispose one to risks for substance use and impact treatment outcomes.

Finally, incorporating patients with lived experiences of methadone treatment in the MOUD clinic can increase provider responsiveness towards patients with OUD. Using the mantra of “nothing about us without us” [48] to guide the clinic operations and development of policies that determine patient’s retention to care can shift thinking towards a framework of PCC. Patients and their families can actively be involved in treatment so that the HCPs can benefit from their experiential knowledge and can ensure patients preferences are considered in the care they receive, which in turn can increase satisfaction with care and improve treatment outcomes [49].

The limitations of this study must be acknowledged. This study was based on self-reported information about how the HCPs and patients interacted to provide care. A policy analysis of the methadone treatment framework was not conducted as part of the study. Since this paper is part of a larger study, the PCC framework was applied in the secondary data analysis and did not inform original instrument development for data collection. Considering this was a small and specific sample size, as with most qualitative research, there is little transferability or generalizability to larger populations or diverse groups. The use of the doctor as a person in the PCC is a misnomer as it assumes that only doctors can engage in PCC. In multidisciplinary settings, it is best to think of HCPs as a person to represent that concept best. We acknowledge that treatment for substance use disorders is complex and requires a judicious appraisal of the patients' needs, best practices in care, and research evidence. Because patients' needs are unique, PCC undoubtedly would look different and unique to each patient. Constant striving to act within the patient’s best interest can perhaps be an excellent guiding principle when providing care within a biomedical framework.

## Conclusion

Principles of PCC are highly applicable within the context of a methadone treatment clinic. Although barriers exist within the current biomedical, restrictive model of care, there are possibilities to reorient treatment to shift towards recovery and a patient-oriented care model without disrupting the treatment framework's philosophy. Such a shift in practice would emphasize retention of patients instead of reinforcing compliance, as strengthening restrictive compliance often leads to low retention. Within a framework of PCC patient expectations to abide by the treatment terms need to be tempered by the determinants of health that they experience. Engaging in a social justice framework can shift HCPs perspectives towards a model of PCC as together these frameworks can foster collaborative care, power sharing, and health equity with all those involved in the care of patients on MOUD. Tension between person centred care that is informed by the need to be human, and task focussed care that is dictated by the protocols and guidelines that prescribe care to patients need to be acknowledged and negotiated.

## Data Availability

Data are available upon request due to privacy or other restrictions.

## Abbreviations

OUD: Opioid use disorders
MOUD: Medications for opioid use disorders
HCPs: Health care providers
PCC: Patient centered care
